# Parasite exosomes-derived circulating sja-miR-61 and sja-miR-7-5p as Novel biomarkers for the detection of *Schistosoma japonicum* infection using TaqMan real-time PCR

**DOI:** 10.1371/journal.pntd.0014368

**Published:** 2026-05-20

**Authors:** Xin-Yue Zhang, Jing-Ping Li, Ju-Lu Lu, Cong-Jin Mei, Ying-Ying Yang, Pan-Pan Dong, Chuan-Xin Yu, Li-Jun Song

**Affiliations:** National Health Commission Key Laboratory of Parasitic Disease Control and Prevention, Jiangsu Provincial Key Laboratory on Parasite and Vector Control Technology, Jiangsu Provincial Medical Key Laboratory, Jiangsu Institute of Parasitic Diseases, Wuxi, China; Umaru Musa Yar’Adua University, NIGERIA

## Abstract

Schistosomiasis, a zoonotic parasitic disease caused by schistosome infection, remains a serious public health concern. However, diagnostic methods with both high sensitivity and specificity or early diagnosis for detecting schistosome infection are still lacking. This study aimed to evaluate the diagnostic potential of parasite-derived exosomes miRNAs in *Schistosoma japonicum* (*S. japonicum*) infection. Exosomes were isolated and purified from adult worms and eggs of *S. japonicum* using ultracentrifugation. Nine highly abundant miRNAs were identified in these exosomes through small RNA sequencing. TaqMan probe-based reverse transcription real-time PCR (RT-qPCR) revealed that serum levels of all nine parasite-derived miRNAs were significantly elevated in infected mice at different time points post-infection. Compared with healthy controls, infected patients exhibited significantly elevated serum levels of sja-miR-61 and sja-miR-7-5p. Receiver operating characteristic (ROC) analysis indicated that the area under the curve (AUC) values for serum sja-miR-61 and sja-miR-7-5p were 0.8750 and 0.8375, respectively, surpassing those of the other miRNAs examined in human serum. The sensitivity and specificity for detecting these miRNAs in human serum were 85.00% and 85.00% for sja-miR-61, and 70.00% and 80.00% for sja-miR-7-5p. When both miRNAs were detected in combination, sensitivity increased to 95.00% with a specificity of 75.00%. In mice, the sensitivity and specificity for detecting sja-miR-7-5p at 2 weeks post-infection were 73.33% and 86.67%, improving to 93.33% and 93.33% at 6 weeks. For sja-miR-61, sensitivity and specificity were 93.33% and 80.00% at 2 weeks, reaching 100.00% and 100.00% at 6 weeks. Serum levels of sja-miR-61 and sja-miR-7-5p dropped to undetectable levels two weeks after praziquantel treatment, underscoring their utility in monitoring treatment response. Furthermore, measurement of sja-miR-61 and sja-miR-7-5p in mice with varying infection intensities showed rising serum levels corresponding to increased worm burdens, with a minimum detection threshold equivalent to an infection with 5 cercariae, highlighting the high sensitivity of this approach for detecting low-level infections in mice. Collectively, the detection of these miRNAs in patient or mouse serum using TaqMan RT-qPCR exhibits good sensitivity and specificity, along with considerable value for early diagnosis and treatment monitoring. sja-miR-61 and sja-miR-7-5p represent promising biomarkers for the diagnosis of schistosomiasis japonica.

## Author summary

The diagnosis of schistosomiasis japonica remains challenging due to a lack of sensitive and specific tests. We identified that sja-miR-61 and sja-miR-7-5p, derived from parasite exosomes, serve as excellent circulating biomarkers for *Schistosoma japonicum* (*S. japonicum*) infection. In human patients, both miRNAs were significantly elevated in serum and served as effective diagnostic biomarkers. Whereas the individual biomarkers sja-miR-61 and sja-miR-7-5p showed 85.00% sensitivity/specificity (AUC = 0.8750) and 70.00% sensitivity/80.00% specificity (AUC = 0.8375) respectively using TaqMan RT-qPCR, a combined test achieved a markedly superior performance, detecting infection with 95.00% sensitivity and 75.00% specificity. In mice, high sensitivity (100%) and specificity (80%) of the combined test were achieved as early as 2 weeks post-infection. Serum miRNA levels became undetectable within two weeks after praziquantel (PZQ) therapy, allowing for rapid assessment of cure. The assay can detect infections with as few as 5 cercariae, addressing a critical need in low-endemic areas. In conclusion, the detection of serum sja-miR-61 and sja-miR-7-5p via TaqMan RT-qPCR provides a sensitive and specific method for diagnosing schistosomiasis japonica, with significant utility for early diagnosis, treatment efficacy monitoring, and surveillance.

## Introduction

Schistosomiasis is a major zoonotic parasitic disease endemic in tropical and subtropical regions. Affecting over 70 countries, it places nearly 800 million people at risk and accounts for more than 70 million disability-adjusted life years (DALYs) lost annually [1,2]. The primary species infecting humans include *Schistosoma mansoni* (*S. mansoni*), *S. japonicum*, and *S. haematobium*. *S. japonicum*, found mainly in East and Southeast Asia—such as China, Japan, the Philippines, and Indonesia—is notable for its high daily egg output, reaching up to 3000 eggs per adult worm [3]. A substantial proportion of these eggs lodge in the host liver, triggering granulomatous inflammation and fibrosis that may progress to portal hypertension, hepatosplenomegaly, ascites, and in advanced cases, to cirrhosis and liver cancer, with severe clinical consequences [4,5]. As control measures advance and infection rates decline, schistosome infections are increasingly characterized by low intensity, complicating accurate diagnosis. Thus, developing highly sensitive and specific methods for early detection is essential to prevent disease progression and curb transmission.

The current diagnostic gold standard for schistosomiasis japonica relies on parasitological techniques, primarily the Kato-Katz thick smear and miracidium hatching test. However, these methods exhibit limited sensitivity, particularly in early and low-intensity infections, with false-negative rates as high as 40–50%. They are also susceptible to operator error and require experienced personnel [5]. Immunoassays targeting schistosome circulating antigens offer a means to detect active infection [6], yet these antigens are often present at low concentrations in serum, challenging to measure accurately, and may cross-react, leading to reduced specificity. Alternatively, detection of host antibodies against schistosome antigens is widely used [7], but seroconversion occurs relatively late, limiting utility for early diagnosis, and antibodies may persist after cure, complicating the distinction between past and current infection.

Nucleic acid-based methods have gained attention for their high sensitivity and rapid turnaround, contributing significantly to advances in schistosomiasis diagnostics [8–11]. The performance of nucleic acid tests hinges on the selection of specific and reproducible molecular targets. Although several schistosome DNA sequences have been evaluated for this purpose [12,13], research focused on early infection remains limited, and some existing protocols are relatively complex to implement [14]. Therefore, there is a clear need to develop simpler, more sensitive, and specific diagnostic tools capable of detecting early-stage infection.

Exosomes, identified in cultures of lymphocytes and dendritic cells, are small (30–150 nm in diameter), lipid bilayer–enclosed extracellular vesicles secreted by various cell types [15]. They are detectable in multiple body fluids, including blood, urine, lymph, cerebrospinal fluid, bile, and saliva [16]. Exosome‑encapsulated miRNAs have emerged as promising diagnostic biomarkers owing to their high specificity, sensitivity, species specificity [17], and stability under diverse conditions [18]. Specific profiles of circulating exosome miRNAs enable early detection of several cancers, highlighting their potential in tumor diagnosis and prognosis [19,20]. For example, elevated levels of miR‑212‑5p, miR‑1248, and miR‑1250‑5p in hepatocellular carcinoma (HCC), when combined with clinical parameters such as gender and cirrhosis, yield high diagnostic specificity and sensitivity [21]. Similarly, plasma exosome miRNA‑30a and miRNA‑192 aid in diagnosing alcoholic hepatitis [22], while serum exosome miR‑195‑3p shows promise as a biomarker for osteosarcoma [23].

MiRNAs are also abundantly present in parasite‑derived exosomes [24–27]. For instance, next‑generation sequencing of *S. mansoni* adult worm exosomes identified 143 miRNAs, among which 25 were confirmed by qPCR to be elevated in sera from infected mice [28]. In *S. japonicum*–infected rabbits, four schistosome‑specific miRNAs (sja‑miR‑3479, sja‑miR‑10, sja‑miR‑3096, and sja‑miR‑8185) were detected in serum [29], and sja‑miR‑277 and sja‑miR‑3479‑3p were identified in infected mice, suggesting a correlation with infection and pathology [30]. However, studies on the diagnostic utility of parasite‑derived exosome miRNAs remain limited, and no highly sensitive and specific parasite miRNA markers have been established for schistosomiasis diagnosis [31].

In this study, exosomes were isolated from secretions and excretions of *S. japonicum* adult worms and eggs. Small RNA sequencing revealed a set of highly abundant miRNAs derived from these parasite exosomes. Their expression was preliminarily verified in a mouse model using TaqMan probe-based reverse transcription real-time PCR (RT-qPCR). Subsequent evaluation in both patient and mouse sera demonstrated that detection of circulating sja‑miR‑61 and sja‑miR‑7‑5p offers high sensitivity and specificity. Further analysis confirmed the value of these parasite‑derived miRNAs for monitoring treatment efficacy and identified sja‑miR‑61 as a potential biomarker for early detection. This study provides a scientific foundation for the discovery of diagnostic and early‑detection biomarkers for schistosomiasis, and supports future development of field‑applicable diagnostic kits.

## Materials and methods

### Ethics statement

All animals including mice and rabbits procedures followed the Guidelines for the Ethical Review of Experimental Animal Welfare (GB/T 35892–2018) and were approved by the Ethics Review Committee of Jiangsu Institute of Parasitic Diseases (Approved numbers: JIPD-2023–002). Schistosomiasis patients and healthy individuals with provided informed consent followed the protocols approved by the Ethics Review Committee of Jiangsu Institute of Parasitic Diseases [32].

### Study design

**Fig 1:**
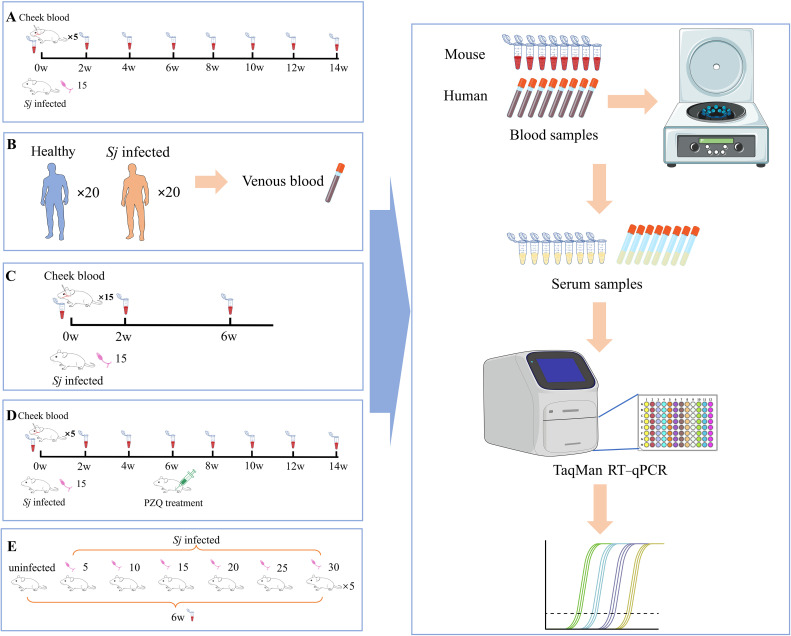
Flowchart of serum sample collection and study design. (A) Collection of serum samples from mice at different time points post-*S. japonicum* infection. **(B)** Collection of serum samples from *S. japonicum*-infected patients and healthy individuals. **(C)** Collection of serum samples from mice with early *S. japonicum* infection. **(D)** Collection of serum samples from mice pre- and post-praziquantel treatment. **(E)** Collection of serum samples from mice with different infection intensities.

The detection efficacy of miRNAs was evaluated in both human patients and mouse models of *Schistosoma japonicum* infection using the TaqMan RT-qPCR method [33,34] (Fig 1). First, to analyze the dynamics of highly abundant exosome miRNAs during infection, five C57BL/6J mice were percutaneously infected with 15 ± 1 cercariae each [35,36], which were released from schistosome-infected positive snails, provided by the Snail Room of Jiangsu Institute of Parasitic Diseases. Blood was collected from the facial vein before infection (0 weeks, w) and at 2, 4, 6, 8, 10, 12, and 14 w post-infection (Fig 1A). For validation in human subjects, serum samples were obtained from 20 schistosomiasis patients (confirmed by fecal egg positivity) in Hubei Province, China, and from 20 healthy controls provided by the Health Management Center of Jiangsu Institute of Parasitic Diseases. All samples were aliquoted for subsequent detection (Fig 1B). To assess the potential of parasite-derived miRNAs for early diagnosis, serum was collected from an additional 15 mice at 2 w and 6 w post-infection (Fig 1C). In a treatment monitoring experiment, five infected mice were administered PZQ (Sigma, St. Louis, Missouri, USA), dissolved in a 1% carboxymethyl cellulose solution, 250 mg/kg/day for 3 days by oral gavage at 6 w. Blood was drawn before infection, at 2 w and 4 w during infection, and at 0, 2, 4, 6, and 8 w after treatment (corresponding to total time points of 6–14 w), which was kept at 4 °C overnight, and serum, separated at 2,500 × *g* for 5 minutes, was used for miRNA analysis (Fig 1D). Finally, to evaluate miRNA performance in low-intensity infections, 35 mice were randomly assigned to seven groups (n = 5) and infected with 0, 5 ± 1, 10 ± 1, 15 ± 1, 20 ± 1, 25 ± 1, or 30 ± 1 cercariae. At 6 w post-infection, blood was collected via the facial vein and processed for serum. Adult worms were then recovered by portal vein perfusion with anticoagulant saline (0.3% sodium citrate, 0.7% NaCl) and counted (Fig 1E).

### Exosome preparation

Six New Zealand White rabbits (1.5–2.5 kg, conventional grade) were purchased from SPF Biotechnology Co., Ltd. (Beijing, China). Each rabbit was percutaneously infected with 1,500 *S. japonicum* cercariae, which were shed from infected *Oncomelania hupensis* snails provided by the Snail Department of the Jiangsu Institute of Parasitic Diseases. At 42 days post-infection, adult worms were perfused from the superior mesenteric vein using anticoagulant saline (0.3% sodium citrate, 0.7% NaCl), and the liver was excised.

The harvested 200 pairs of adult worms were washed three to five times with 200 mL of PBS and cultured in 40 mL of RPMI 1640 medium (VivaCell Biosciences, Shanghai, China) in a 90 mm culture dish supplemented with 10% exosome-depleted fetal bovine serum, 100 U/mL penicillin (Solarbio, Beijing, China), and 100 µg/mL streptomycin (Solarbio, Beijing, China), in a 5% CO₂ incubator for 48 hours. The excised livers were minced and homogenized in pre-chilled 1.2% NaCl solution using an electric homogenizer. The homogenate was diluted approximately 10-fold with additional cold 1.2% NaCl solution and sequentially filtered through 80-, 150-, and 200-mesh sieves. The resulting filtrate was collected in a 300-mesh nylon bag, and after water was removed, the eggs retained in the bag were harvested. The egg pellet was resuspended in 100 mL ice-cold 1.2% NaCl solution and centrifuged at 100 × *g* for approximately 1 minute. The supernatant and upper white layer were discarded, and the lower yellow layer containing the eggs was collected. The eggs were then digested with 0.05% pre-chilled collagenase IV (Sigma, St. Louis, Missouri, USA) in PBS at 37 °C for 1.5 hours [37], washed with RPMI 1640 medium containing 10% exosome-depleted serum (VivaCell Biosciences, Shanghai, China), and collected by centrifugation at 1,500 × *g*, then counted under a microscope. Finally, 5 × 10^4^ of eggs were incubated in 40 mL of RPMI 1640 medium in a 90 mm culture dish with 10% exosome-depleted serum in a 5% CO₂ incubator for 48 hours.

All the culture supernatants (40 mL) from both adult worms and eggs (after 48-hour culture in exosome-depleted serum medium) were collected. Exosomes were isolated using sequential ultracentrifugation: first at 300 × *g* for 10 minutes at 4 °C to remove cells, followed by 1,500 × *g* for 30 minutes to pellet debris. The supernatant was then subjected to ultracentrifugation at 120,000 × *g* for 70 minutes. The resulting exosome pellet was collected and stored at –80 °C. Three replicate samples were taken from a single pool of adult and egg exosomes.

### Exosome characterization and small RNA sequencing

Collected adult worm and egg-derived exosomes were shipped on dry ice to Shanghai OE Biotech Co., Ltd. Morphology was examined using a HT7700 transmission electron microscope (TEM) at an accelerating voltage of 100 kV. Particle size distribution was determined using a NanoFCM N30E.

Three replicate samples each of adult worm and egg exosomes were sent to Shanghai OE Biotech Co., Ltd. for small RNA sequencing analysis on an Illumina high-throughput next-generation sequencing platform to identify miRNA profiles. Briefly, total RNA was extracted using the mirVana miRNA Isolation Kit (Ambion, Texas, USA) following the manufacturer’s protocol. RNA quantity and integrity were assessed using a Nanodrop 2000 (Thermo Fisher Scientific, Massachusetts, USA) and an Agilent 2100 Bioanalyzer (Agilent Technologies, California, USA), respectively. For each sample, 1 µg of total RNA was used to construct small RNA libraries using the NEBNext Small RNA Library Prep Set for Illumina (NEB, Massachusetts, USA). Adapters were ligated to both ends of the RNA, followed by reverse transcription to cDNA and PCR amplification. PCR products (~140–160 bp) were size-selected and purified by agarose gel electrophoresis to generate the final small RNA libraries. Library quality was verified using the Agilent 2100 Bioanalyzer before sequencing on the Illumina Novaseq 6000 platform. Raw sequencing data (RawData/RawReads) generated from base calling were processed to remove low-quality reads, reads containing 5’ primer contaminants or poly(A) tails, reads without 3’ adapters or insert tags, and reads shorter than 15 nt or longer than 41 nt, resulting in clean reads. Raw sequencing data were submitted to the NCBI SRA database (Accession number: PRJNA1342957).

For small RNA analysis, clean reads were first analyzed for length distribution against the reference genome. Bowtie software was used to align sequences against the Rfam v10.1 database (http://www.sanger.ac.uk/software/Rfam) to annotate and filter out rRNA, scRNA, cis-regulatory elements, snRNA, tRNA, etc. Subsequent alignments were performed sequentially against cDNA sequences, the Repbase database for repetitive sequences, and the miRBase database (http://www.mirbase.org/) to remove degraded transcript sequences, repeats, and to identify and annotate known miRNAs. Expression patterns of known miRNAs across samples were analyzed. Novel miRNAs were predicted from the remaining unannotated reads using miRDeep2 software, which identifies potential miRNA precursors based on hairpin structures and known miRNAs in miRBase, predicting mature and star sequences.

### SYBR Green RT-qPCR

RNA was extracted from 100 mg of cercariae, adult worms, and eggs using a tissue RNA extraction kit (SparkJade, Jinan, China), and 500 ng of RNA was reverse-transcribed into cDNA using a miRNA 1st Strand cDNA Synthesis Kit (Vazyme, Nanjing, China). The miRNA at the cercariae, adult worm, and egg stages were amplified using miRNA Unimodal SYBR qPCR Master Mix (Vazyme, Nanjing, China), with amplification primers listed in S1 Table. The reaction conditions were: 95 °C 30 s; 95 °C 10 s, 60 °C 30 s, 40 cycles. The relative expression levels were calculated by the 2^-ΔΔCt^ method using U6 as a control [38].

### TaqMan RT-qPCR

Total miRNA was extracted from 200 µL of mouse serum or 300 µL of human serum using a Plasma miRNA Extraction Kit (HaiGene, B1804, Harbin, China). Extracted miRNA was reverse transcribed into cDNA using the TaqMan miRNA cDNA Synthesis Kit (Serum/Plasma) (HaiGene, D1803, Harbin, China). Custom-designed HG TaqMan miRNA qPCR Assays (HaiGene, Harbin, China) were used for TaqMan probe-based qPCR amplification of target miRNAs (1,250 ng) from cDNA samples of infected mouse serum, patient serum, and controls. The reaction mixture (20 µL total) contained: 4 µL 5 × Golden HS TaqMan qPCR Mix, 1 µL 10 × miRNA TaqMan Assay, 2.5 µL cDNA template, and 12.5 µL ddH₂O. Thermal cycling conditions were: 95°C for 15 min; 40–45 cycles of 95°C for 10 s and 60°C for 30 s. Amplification and cycle threshold (Ct) calculation were performed using an Applied Biosystems QuantStudio 6 & 7 Pro instrument (Thermo Fisher Scientific, Massachusetts, USA). Samples with Ct values > 36 were excluded. miR-16 was used as the housekeeping gene [39,40]. The amplified products were sent to Sangon Biotech Co., Ltd. (Shanghai, China) for sequencing to confirm their specificity.

### Statistical analysis

Data analysis was performed using GraphPad Prism 9.5. Normally distributed measurement data were expressed as mean ± standard deviation. Group comparisons were conducted using the t-test. To account for inter-plate variability, Ct values for all samples were normalized using the formula: Normalized Ct value of sample miRNA = Raw Ct value of sample miRNA × (Mean Ct value of reference gene in healthy individuals / Mean Ct value of reference gene in the sample). The mean Ct value for each miRNA across all healthy individuals was calculated, and the ΔCt value for each sample was obtained by subtracting the normalized sample Ct value from the mean Ct value of healthy controls. These ΔCt values were used to evaluate the diagnostic performance of each miRNA [31].

Receiver operating characteristic (ROC) curves were constructed using ΔCt values to evaluate the diagnostic performance of microRNAs in discriminating between schistosome-infected patients and healthy controls, as well as between pre- and post-infection mice. The area under the curve (AUC), sensitivity, and specificity were calculated. The optimal cutoff value on each ROC curve was selected as the point yielding the maximum Youden’s index (Youden’s index = Sensitivity + Specificity – 1). In cases where multiple points shared the same Youden’s index, the one with the higher sensitivity was preferred [41,42]. A sample was classified as positive if its ΔCt value was greater than the established cutoff. For combined diagnostic evaluation, a sample was deemed positive if the ΔCt value of either sja-miR-61 or sja-miR-7-5p exceeded its respective cutoff. A *P* value less than 0.05 was considered statistically significant.

## Results

### Morphological characteristics and miRNA composition of exosomes derived from *S. japonicum* adults and eggs

**Fig 2:**
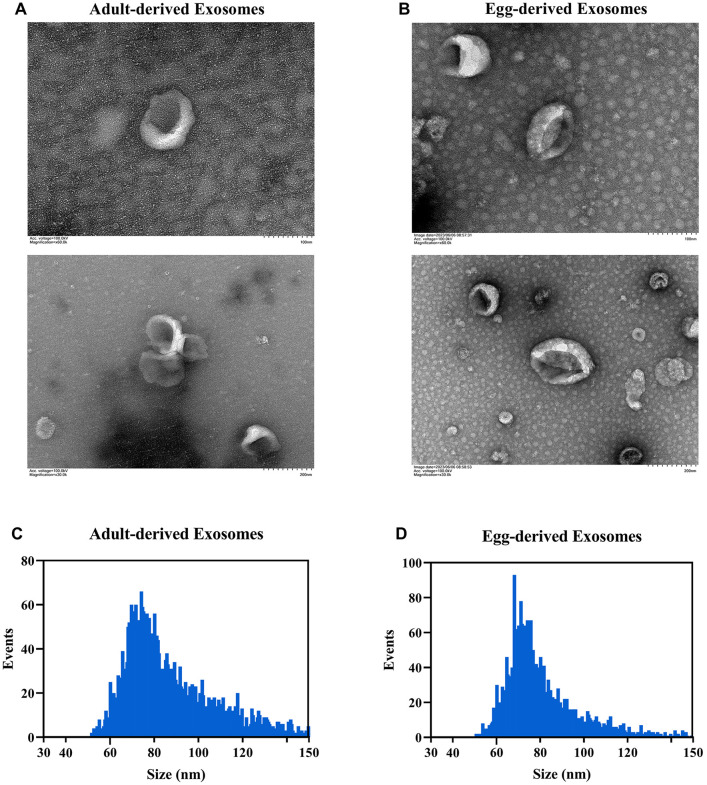
Morphological characteristics of *S. japonicum* adult worm- or egg-derived exosomes under transmission electron microscopy (TEM) and Nanoparticle tracking analysis. A: TEM image of adult worm-derived exosomes; B: TEM image of egg-derived exosomes; C: Size distribution of adult worm-derived exosomes; D: Size distribution of egg-derived exosomes.

Exosomes from *S. japonicum* adult worms and eggs were isolated and purified by ultracentrifugation. TEM analysis revealed that both adult worm-derived and egg-derived exosomes exhibited a cup-shaped, spherical morphology with a double-membrane structure, consistent with typical exosome morphology (Fig 2A, B). Nanoparticle tracking analysis showed particle sizes ranging from 50 to 150 nm, with average sizes of 86.98 nm for adult worm exosomes (Fig 2C) and 82.60 nm for egg exosomes (Fig 2D).

Illumina high-throughput next-generation sequencing was used to determine the miRNA profiles in adult worm and egg exosomes. All identified miRNA sequences and their read counts are provided in S2 and S3 Tables. The top 5 most abundant miRNAs (by read count) in adult worm exosomes were sja-miR-125b, sja-miR-71a, sja-miR-125, sja-miR-61, and sja-miR-36-3p. The top 5 most abundant miRNAs in egg exosomes were sja-miR-36-3p, sja-miR-1, sja-miR-71b-5p, sja-bantam, and sja-miR-7-5p. Notably, sja-miR-36-3p was highly abundant in both adult worm and egg exosomes. Sequences, genomic locations, and read counts for these 9 miRNAs are summarized in Table 1. The expression of these miRNAs in schistosome cercariae, adults, and eggs was detected using the SYBR Green RT-qPCR. The results showed that sja-miR-125b, sja-miR-71a, sja-miR-125, and sja-miR-61 were most highly expressed in the adult stage, whereas sja-miR-36-3p, sja-miR-1, sja-miR-71b-5p, sja-bantam, and sja-miR-7-5p were most highly expressed in the egg stage, consistent with the sequencing results. Furthermore, sja-miR-71a, sja-miR-125, sja-miR-61, sja-miR-1, sja-bantam, and sja-miR-7-5p were also expressed at the cercariae stage (S1 Fig).

**Table 1:** The top 9 miRNAs Expressed in the Exosomes of *S. japonicum* Adults and Eggs.

Small RNA IDs	miRNAs	Genomic locations	Sequence	Size (nt)	Read count (worms)	Read count (eggs)
**MIMAT0010179**	sja-miR-125b	CABF01027351.1: 4363–4440 [-]	UCCCUGAGACUGAUAAUUGCUC	22	336297 ± 62378	2406 ± 1409
**MIMAT0010176**	sja-miR-71a	CABF01007682.1: 3056–3135 [-]	UGAAAGACGAUGGUAGUGAGA	21	106212 ± 2349	47616 ± 1117
**MIMAT0010178**	sja-miR-125	CABF01024723.1：7689–7772 [+]	UCCCUGAGACUGAUAAUUGCU	21	46239 ± 29038	1119 ± 956
**MIMAT0016259**	sja-miR-61	CABF01036171.1: 1214–1356 [+]	UGACUAGAAAGUGCACUCACUU	22	25541 ± 8009	8352 ± 1101
**MIMAT0010178**	sja-miR-36-3p	CABF01004852.1: 272–364 [-]	CCACCGGGUAGACAUUCAUUCGC	23	14981 ± 4295	1613140 ± 77666
**MIMAT0016244**	sja-miR-1	CABF01001061.1: 9–93 [+]	UGGAAUGUGGCGAAGUAUGGUC	22	1995 ± 180	379567 ± 7189
**MIMAT0016260**	sja-miR-71b-5p	CABF01011791.1: 2817–2909 [+]	UGAAAGACUUGAGUAGUGAGACG	23	2388 ± 227	284967 ± 38357
**MIMAT0010177**	sja-bantam	CNUS0000021739:2223–2244	UGAGAUCGCGAUUAAAGCUGGU	22	8243 ± 2038	191476 ± 37687
**MIMAT0016249**	sja-miR-7-5p	CABF01039688.1: 5408–5554 [+]	UGGAAGACUGGUGAUAUGUUGUU	23	4717 ± 423	57962 ± 2181

### Levels of high-abundance parasite-derived exosomal miRNAs in mouse sera at different stages of schistosome infection

**Fig 3:**
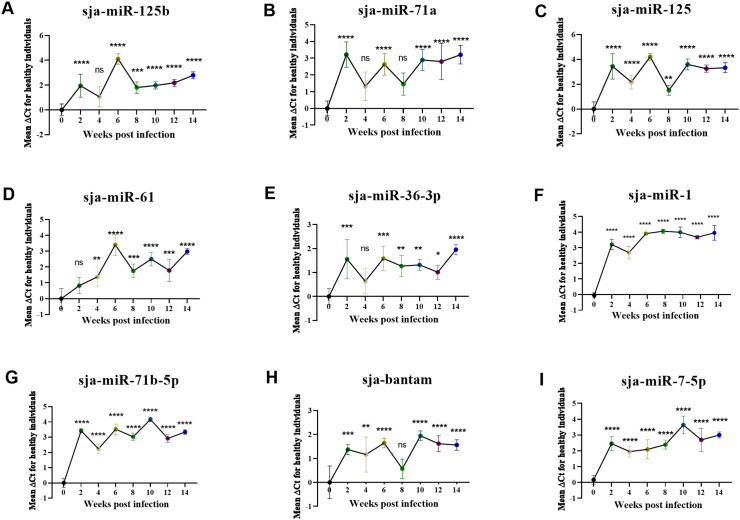
Changes in levels of parasite-derived miRNAs in mouse serum at different time points before and after *S. japonicum* infection. A: sja-miR-125b; B: sja-miR-71a; C: sja-miR-125; D: sja-miR-61; E: sja-miR-36-3p; F: sja-miR-1; G: sja-miR-71b-5p; H: sja-bantam; I: sja-miR-7-5p (Compared with pre-infection, ** *p* < 0.01, *** *p* < 0.001, **** *p* < 0.0001, ns, not significant).

To evaluate the diagnostic potential of these high-abundance miRNAs, specific TaqMan probes were used in RT-qPCR to measure their levels in mouse sera collected pre-infection (0 w) and at 2, 4, 6, 8, 10, 12, and 14 w. Compared to pre-infection levels, all nine miRNAs were significantly elevated in sera from infected mice. The accuracy of the sequence was verified by sequencing the amplified products. The sequencing results were consistent with the 5’ and central sequences of these miRNAs, with 2–3 base inconsistencies at the 3’ end (S2 Fig), which was related to the presence of a 3’ isomiR of miRNA [43]. Among adult worm exosome-derived miRNAs, sja-miR-125b (Fig 3A), sja-miR-125 (Fig 3C), and sja-miR-61 (Fig 3D) peaked before 6 w, with fluctuating but persistently elevated levels thereafter. sja-miR-71a (Fig 3B) peaked as early as 2 w, suggesting high expression during the schistosomula stage. Egg exosome-derived miRNAs—sja-miR-36-3p (Fig 3E), sja-miR-1 (Fig 3F), sja-miR-71b-5p (Fig 3G), sja-bantam (Fig 3H), and sja-miR-7-5p (Fig 3I)—also increased gradually post-infection but peaked later, between 10 and 14 w. This delayed peak likely correlates with egg production commencing around 3 weeks post-infection and subsequent accumulation. These results demonstrate significantly elevated serum levels of all nine miRNAs from 2 w post-infection onwards compared to pre-infection (all *p* < 0.05, Fig 3), indicating their potential diagnostic value.

### Diagnostic performance of TaqMan RT-qPCR for detecting parasite-derived exosomal miRNAs in sera of schistosome-infected patients

**Fig 4:**
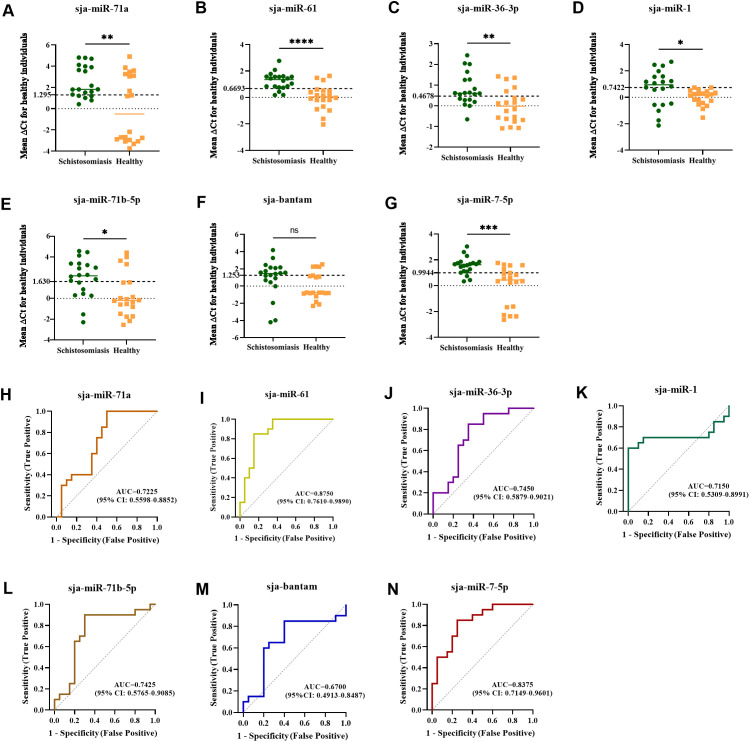
Levels of high-abundance miRNAs in sera of schistosomiasis patients. Comparison of levels between *S. japonicum* patient and healthy control sera for (A) sja-miR-71a, (B) sja-miR-61, (C) sja-miR-36-3p, (D) sja-miR-1, (E) sja-miR-71b-5p, (F) sja-bantam, (G) sja-miR-7-5p. Receiver operating characteristic (ROC) curves for the detection of the 7 miRNAs in patient vs. healthy control sera: (H) sja-miR-71a, (I) sja-miR-61, (J) sja-miR-36-3p, (K) sja-miR-1, (L) sja-miR-71b-5p, (M) sja-bantam, (N) sja-miR-7-5p (* *p* < 0.05, ** *p* < 0.01, *** *p* < 0.001, **** *p* < 0.0001, ns, not significant).

To validate the diagnostic value of these miRNAs in human infection, their levels were measured in human sera using TaqMan RT-qPCR. sja-miR-125 and sja-miR-125b were not amplified in patient sera. After adding these two miRNAs to human serum samples respectively, the corresponding miRNA was amplified, which confirmed that the absence was true (S3 Fig). The remaining seven miRNAs were detected (Fig 4A-G). Compared to healthy controls, the mean ΔCt values for sja-miR-71a (Fig 4A), sja-miR-61 (Fig 4B), sja-miR-36-3p (Fig 4C), sja-miR-1 (Fig 4D), sja-miR-71b-5p (Fig 4E), and sja-miR-7-5p (Fig 4G) were significantly higher in patient sera (all *p* < 0.05). The differences in levels (mean ΔCt) for sja-miR-61 and sja-miR-7-5p between patients and controls were more pronounced than for the other miRNAs. In contrast, the level of sja-bantam (Fig 4F) did not differ significantly between groups (*p* > 0.05).

Diagnostic performance was assessed using ROC curves and AUC analysis. The AUC indicates the ability of a miRNA to discriminate between diseased and healthy populations, with values closer to 1.0 indicating better performance. Analysis showed that sja-miR-61 and sja-miR-7-5p had the highest AUC values, 0.8750 and 0.8375, respectively (Fig 4H-N). Detecting sja-miR-61 in patient serum yielded 17 positives out of 20 (85.00% positivity rate); among 20 healthy controls, 3 were positive (15.00% false positive rate), resulting in 85.00% sensitivity and 85% specificity. For sja-miR-7-5p, 14 out of 20 patients were positive (70.00% positivity rate), and 4 out of 20 controls were positive (20.00% false positive rate), yielding 70.00% sensitivity and 80.00% specificity (Table 2). Combined detection of both miRNAs increased sensitivity to 95.00% with a specificity of 75.00% (Table 2). These findings suggest that sja-miR-61 and sja-miR-7-5p offer good sensitivity and specificity for diagnosing human schistosome infection, and their combined use enhances detection sensitivity.

**Table 2:** Sensitivity and Specificity of TaqMan RT-qPCR for Detection of miRNAs derived from exosomes of *S. japonicum* in Human Sera.

		Test Results Among Patients		Test Results Among Healthy			
miRNA	Cut-off	Positive	Negative	Positive	Negative	Sensitivity (95% CI)	Specificity (95% CI)
**sja-miR-71a**	1.295	15	5	8	12	75.00 (53.13-88.81)	60.00 (38.66-78.12)
**sja-miR-61**	0.6693	17	3	3	17	85.00 (63.96-94.76)	85.00 (63.96-94.76)
**sja-miR-36-3p**	0.4678	13	7	5	15	65.00 (43.29-81.88)	75.00 (53.13-88.81)
**sja-miR-1**	0.6295	13	7	2	18	65.00 (43.29-81.88)	90.00 (69.90-98.22)
**sja-miR-71b-5p**	0.2006	18	2	6	14	90.00 (69.90-98.22)	70.00 (48.10-85.45)
**sja-bantam**	1.253	12	8	4	16	60.00 (38.66-78.12)	80.00 (58.40-91.93)
**sja-miR-7-5p**	1.270	14	6	4	16	70.00 (48.10-85.45)	80.00 (58.40-91.93)
**Total** ^*^		19	1	5	15	95.00	75.00

*Samples with a delta threshold cycle above the cutoff for any of sja-miR-61 or sja-miR-7-5p were considered positive [31].

### Detection of sja-miR-61 and sja-miR-7-5p in sera of mice at 2 and 6 weeks post-infection demonstrates value for early diagnosis

Given the promising sensitivity and specificity observed in human studies, we further investigated the potential of sja-miR-61 and sja-miR-7-5p for early diagnosis. Their levels were measured in mouse sera pre-infection and at 2 and 6 w using TaqMan RT-qPCR. ROC curve analysis based on ΔCt values was used to evaluate diagnostic performance.

At 2 weeks post-infection, detection of sja-miR-61 yielded 14 positives out of 15 infected mice (93.33% positivity rate), while sja-miR-7-5p detection yielded 11 positives (73.33% positivity rate) (Table 3). At 6 weeks post-infection, sja-miR-61 was detected in all 15 infected mice (100.00% positivity), and sja-miR-7-5p was detected in 14 out of 15 mice (93.33% positivity) (Table 4).

**Table 3:** Sensitivity and Specificity of TaqMan RT-qPCR for Detection of miRNAs derived from exosomes of *S. japonicum* in Mice Sera at 2 weeks post-infection.

		Test Results Among Infected mice		Test Results Among Healthy mice			
miRNA	Cut-off	Positive	Negative	Positive	Negative	Sensitivity (95% CI)	Specificity (95% CI)
**sja-miR-61**	0.6389	14	1	3	12	93.33 (70.18-99.66)	80.00 (54.81-92.95)
**sja-miR-7-5p**	0.4900	11	4	2	13	73.33 (48.05-89.10)	86.67 (62.12-97.63)
**Combined Detection** ^**a**^		15	0	3	12	100.00	80.00

^a^Samples with a delta threshold cycle above the cut-off for any of the miRNAs were considered positive [31].

**Table 4:** Sensitivity and Specificity of TaqMan RT-qPCR for Detection of miRNAs derived from exosomes of *S. japonicum* in Mice Sera at 6 weeks post-infection.

		Test Results Among Infected mice		Test Results Among Healthy mice			
miRNA	Cut-off	Positive	Negative	Positive	Negative	Sensitivity (95% CI)	Specificity (95% CI)
**sja-miR-61**	0.9855	15	0	0	15	100.00 (79.61-100.00)	100.00 (79.61-100.00)
**sja-miR-7-5p**	0.8000	14	1	1	14	93.33 (70.18-99.66)	93.33 (70.18-99.66)
**Combined Detection** ^**a**^		15	0	1	14	100.00	93.33

^a^Samples with a delta threshold cycle above the cut-off for any of the miRNAs were considered positive [31].

**Fig 5:**
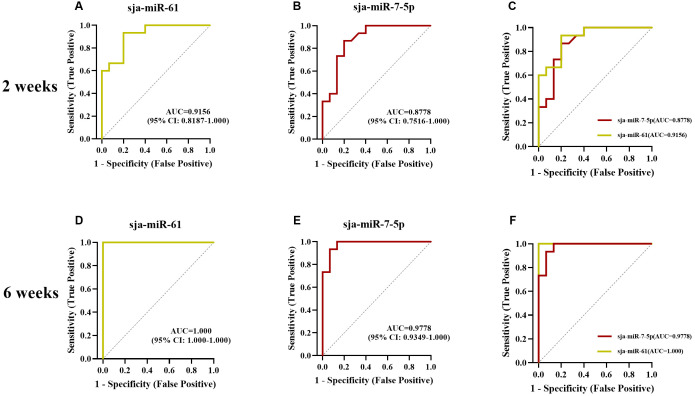
Receiver operating characteristic (ROC)curves for detection of sja-miR-61 and sja-miR-7-5p in mouse serum at 2 and 6 weeks post-infection. (A) ROC curve for sja-miR-61 detection at 2 weeks post infection; **(B)** ROC curve for sja-miR-7-5p detection at 2 weeks post infection; **(C)** Comparison of ROC curves for combined detection of sja-miR-7-5p and sja-miR-61 at 2 weeks post infection; **(D)** ROC curve for sja-miR-61 detection at 6 weeks post infection; **(E)** ROC curve for sja-miR-7-5p detection at 6 weeks post infection; **(F)** Comparison of ROC curves for combined detection of sja-miR-7-5p and sja-miR-61 at 6 weeks post infection.

ROC analysis of the 2-week samples yielded an AUC of 0.9156 (95% CI: 0.8187-1.000, *p* < 0.0001) for sja-miR-61, with an optimal cut-off of 0.6389 demonstrating 93.33% sensitivity and 80.00% specificity. In contrast, sja-miR-7-5p showed an AUC of 0.8778 (95% CI: 0.7516-1.000, *p* ＜ 0.01), for which a cut-off of 0.4900 provided 73.33% sensitivity and 86.67% specificity (Table 3). At the 6-week time point, sja-miR-61 achieved a perfect AUC of 1.000 (95% CI: 1.000-1.000, *p* ＜ 0.0001) with a cut-off of 0.9855, resulting in 100.00% sensitivity and specificity. Similarly, sja-miR-7-5p exhibited an AUC of 0.9778 (95% CI: 0.9349-1.000, *p* ＜ 0.0001), and a cut-off of 0.8000 yielded 93.33% for both sensitivity and specificity (Fig 5) (Table 4).

These results establish sja-miR-61 as a valuable biomarker for the early diagnosis of schistosome infection, demonstrating robust sensitivity and specificity as early as 2 weeks post-infection. Furthermore, a combined detection approach utilizing both miRNAs increased the sensitivity to 100.00% (with a specificity of 80.00%) for 2-week samples and to 100.00% (with a specificity of 93.33%) for 6-week samples, thereby confirming that a dual-miRNA strategy can significantly enhance diagnostic sensitivity.

### Value of Detecting Serum sja-miR-61 and sja-miR-7-5p Levels for Assessing PZQ Treatment Efficacy in Infected Mice

**Fig 6:**
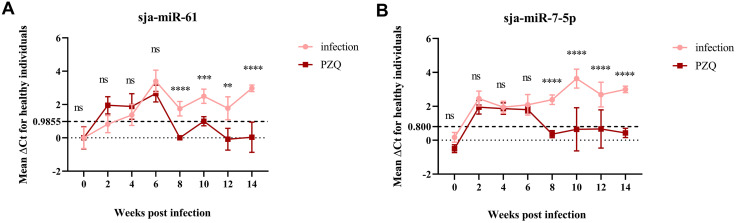
Decline and negative conversion of serum levels of sja-miR-61 and sja-miR-7-5p in *S. japonicum*-infected mice after praziquantel treatment. Mice were treated with praziquantel at 6 weeks post infection in PZQ group. A: sja-miR-61; B: sja-miR-7-5p (Infection compared with PZQ group, ** *p* ＜ 0.01, *** *p* ＜ 0.001, **** *p* ＜ 0.0001, ns, not significant).

Levels of sja-miR-61 and sja-miR-7-5p were measured by TaqMan RT-qPCR in sera from mice collected pre- and post-PZQ treatment (starting at 6 w). Using the ΔCt cut-off values established for 6 w pre-treatment samples as the positivity standard, results showed that both miRNA levels dropped to negative levels by 2 weeks post-treatment. The negative conversion rate was 100.00% for both sja-miR-61 and sja-miR-7-5p. In contrast, miRNA levels remained high in untreated control mice, with statistically significant differences persisting until the end of the observation period at 14 w (Fig 6A-B). These results suggest that detecting serum levels of these schistosome exosome-derived miRNAs holds promise for evaluating the efficacy of PZQ treatment.

### Circulating serum sja-miR-61 and sja-miR-7-5p as biomarkers for low-intensity schistosome infection in mice

**Fig 7:**
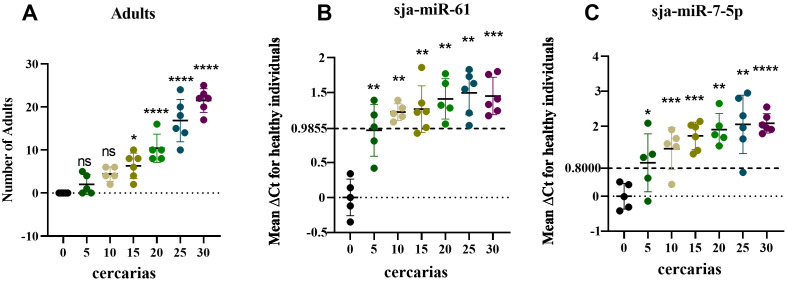
Levels of circulating serum sja-miR-61 and sja-miR-7-5p in mice with different *S.* *japonicum* infection intensities. (A) Adult worm burdens in mice 6 weeks post-infection with 0, 5, 10, 15, 20, 25, or 30 cercariae. **(B)** Levels of sja-miR-61 in sera of mice with different infection intensities. **(C)** Levels of sja-miR-7-5p in sera of mice with different infection intensities. (Compared with pre-infection, * *p* < 0.05, ** *p* < 0.01, *** *p* < 0.001, **** *p* < 0.0001, ns, not significant).

To further validate the diagnostic value of sja-miR-61 and sja-miR-7-5p for low-intensity infections, mice were infected with 5 ± 1, 10 ± 1, 15 ± 1, 20 ± 1, 25 ± 1, or 30 ± 1 cercariae. Sera were collected at 6 weeks post-infection, followed by perfusion of adult worms. The mean adult worm counts were 2.0 ± 2.1, 4.4 ± 1.5, 6.3 ± 2.7, 10.4 ± 2.9, 16.8 ± 4.5, and 21.5 ± 2.5 worms, respectively (Fig 7A). TaqMan qPCR analysis revealed gradually increasing serum levels of both sja-miR-61 and sja-miR-7-5p with higher infection intensities. Applying the ΔCt cut-off values determined for 6 weeks post-infection, at the lowest infection level (5 cercariae), detection of sja-miR-7-5p and sja-miR-61 each yielded 3 positives out of 5 mice (60.00% positivity rate). In the 10-cercariae group, sja-miR-7-5p was detected in 4 out of 5 mice (80.00% positivity), while sja-miR-61 was detected in all 5 mice (100.00% positivity). With the exception of one mouse in the 25-cercariae group testing negative for sja-miR-7-5p and one mouse in the 15-cercariae group testing negative for sja-miR-61, all other mice in the 15, 20, 25, and 30 cercariae groups tested positive for both miRNAs. Circulating sja-miR-7-5p and sja-miR-61 were detectable in mice infected with just 5 cercariae, with levels significantly different from pre-infection levels (*p* < 0.05) (Fig 7B, C). This indicates that sja-miR-61 and sja-miR-7-5p can serve as nucleic acid biomarkers for diagnosing low-intensity schistosome infections in mice. However, when the infection level observed in mice is translated to humans based on worm biomass per millilitre of blood, it corresponds to a moderate-to-heavy rather than a low-level infection. This underscores the need for caution when extrapolating data across species.

## Discussion

Current challenges in the diagnosis of schistosome infection primarily involve the detection of low-intensity infections and achieving timely diagnosis [44]. Parasite-derived exosome miRNAs present benefits including species specificity and stability [45], while nucleic acid detection methods are recognized for high sensitivity and rapid turnaround [46]. Consequently, exosome miRNAs are increasingly being investigated as molecular biomarkers for disease diagnosis, including early detection. Our study screened high-abundance miRNAs in exosomes derived from adult worms and eggs of *S. japonicum*, identifying parasite-derived exosome sja-miR-61 and sja-miR-7-5p as molecular markers for diagnosing schistosomiasis and monitoring drug treatment efficacy, with sja-miR-61 showing particular promise for early diagnosis.

Schistosomes reside within the host’s circulatory system. MiRNAs expressed by adult worms or eggs can be packaged into exosomes, secreted into the host bloodstream, and circulate systemically, rendering them detectable and potentially effective molecular targets for diagnosis. In this study, exosomes were isolated from *S. japonicum* adults and eggs. Small RNA sequencing identified five highly abundant miRNAs in adult worm-derived exosomes (Table 1). Consistent with findings by Lihui Zhu et al. [47], sja-miR-125b and sja-miR-61 were highly expressed in adult worm exosomes; additionally, sja-miR-71a, sja-miR-125, and sja-miR-36-3p were also present at high levels. In egg-derived exosomes, sja-miR-36-3p, sja-miR-1, sja-miR-71b-5p, sja-bantam, and sja-miR-7-5p were highly abundant. To evaluate the diagnostic utility of these miRNAs, specific TaqMan probes were designed and synthesized for all nine candidates. A TaqMan RT-qPCR assay targeting these *S. japonicum* adult and egg exosome-derived miRNAs was established. TaqMan Real-time PCR amplification of serum samples from infected mice revealed significantly elevated levels of all nine high-abundance miRNAs starting from 2 weeks post-infection. However, the time to peak serum levels varied among these miRNAs. miRNAs highly abundant in egg exosomes peaked later than those derived from adult worm exosomes. This is likely because after cercariae infect the host, they migrate and mature into adults residing in the mesenteric veins, a process taking 4–6 weeks [48,49]. Adult worms then begin egg production. Animal studies indicate that fecal egg counts in C57BL/6 mice infected with *S. japonicum* start to rise around 5 weeks, stabilizing at a high level after 8 weeks [50], with peak fecal egg output typically reached at 7–8 weeks [51], and total liver egg burden peaking around 10 weeks [52]. This suggests that levels of egg-derived miRNAs in the host correlate with the timing and quantity of egg production. Minor discrepancies in trends observed in our data may be attributed to miRNA degradation in serum or individual variations among mice. Collectively, these results support the potential diagnostic value of detecting miRNAs derived from adult worm or egg exosomes for schistosome infection.

ROC curve analysis was performed to evaluate the diagnostic potential of these miRNAs in patient sera. ROC curves illustrate the relationship between the true positive rate (sensitivity) and the false positive rate (1 − specificity) across a range of decision thresholds and serve as a fundamental tool for assessing diagnostic accuracy. The selection of an appropriate threshold is critical for optimizing the balance between sensitivity and specificity [53]. In this study, the point on the ROC curve corresponding to the maximum likelihood ratio yielded low sensitivity (<60.00%), rendering it unsuitable as a screening cut-off. Therefore, an alternative threshold was chosen based on a relatively high likelihood ratio and the maximum Youden’s index, with priority given to sensitivity in cases where Youden’s index was equal [41,42,54]. Using this criterion, sja-miR-7-5p demonstrated a sensitivity of 70.00% and specificity of 80.00%, whereas sja-miR-61 achieved both sensitivity and specificity of 85.00%. These values are higher than those reported for sja-miR-2b-5p and sja-miR-2c-5p in human schistosome infection (66%/68% and 55%/80%, respectively) [55]. Combined detection of sja-miR-7-5p and sja-miR-61 resulted in 95.00% sensitivity and 75.00% specificity for diagnosing human infection, indicating that a multi-miRNA strategy can enhance sensitivity, though further improvement in specificity is needed.

Schistosome infections involve numerous reservoir hosts, including large livestock such as cattle and sheep, as well as over 40 mammalian species including rodents. With ecological restoration, wildlife populations—particularly wild rodents—are recovering or increasing [56,57]. The persistence of schistosomiasis in natural foci is complicated by the presence of reservoir hosts. Wildlife and intermediate host snails can become key sources of transmission, underscoring the importance of monitoring schistosome infection in wild animals, especially rodents, for early identification of reservoir hosts and implementation of targeted control measures. Such efforts are essential for the overall containment of schistosomiasis transmission. Few studies have evaluated the diagnostic performance of parasite-derived miRNAs for detecting schistosome infection in mice. In this study, at this early time point of 2 weeks post-infection, sja-miR-61 exhibited high sensitivity (93.33%). Combined detection of both serum sja-miR-61 and sja-miR-7-5p further improved diagnostic sensitivity to 100.00%. By 6 weeks post-infection, both sja-miR-61 and sja-miR-7-5p reached sensitivities and specificities between 90.00% and 100.00%, indicating even stronger diagnostic performance in rodents. Notably, sja-miR-61 showed high sensitivity as early as 2 weeks post-infection, highlighting its promise as an early diagnostic marker in mice. However, the diagnostic value in humans at an early stage still needs to be confirmed among individuals infected at this stage.

Immunological assays such as ELISA, which detect antibodies against antigens like KLH or SEA, can achieve 90.00%–100.00% sensitivity but often suffer from lower specificity [52,58–61]. Moreover, serological detection of anti-schistosome antibodies cannot differentiate between current and past infections [62]. In contrast, detection of circulating parasite-derived miRNAs in serum offers higher sensitivity and specificity. DNA-based methods also demonstrate high diagnostic performance; for example, real-time PCR detection in goat serum reported 98.74% sensitivity and 100% specificity [63–65]. Our findings were consistent with these results and underscore the advantages of nucleic acid-based diagnostics for schistosomiasis.

PZQ remains the primary therapeutic agent for schistosomiasis. However, after decades of widespread use, reduced susceptibility to PZQ has been documented in *S. mansoni* [66]. Although confirmed PZQ resistance has not yet been reported in *S. japonicum*, monitoring therapeutic efficacy remains essential. Previous study has shown that schistosome-derived miRNAs in serum exosomes of infected individuals become undetectable by 32 weeks post-treatment [31]. Similarly, our study observed a decline in serum levels of sja-miR-7-5p and sja-miR-61 following praziquantel administration, suggesting that schistosome-derived miRNAs in serum can serve as an indicator of the efficacy of PZQ treatment. However, there are some differences. The previous study reported that the miRNAs decline slowly after PZQ treatment, remaining low positive at 11 weeks after treatment and turning negative after approximately 32 weeks [31]. Our study, however, found that sja-miR-61 and sja-miR-7-5p declined rapidly after PZQ treatment, turning negative within two weeks. One possible reason for this discrepancy is that the previous study involved treating human-infected individuals with 60 mg/kg of PZQ, whereas this study used a mouse model treated with 250 mg/kg of PZQ. Compared to human studies, the mouse model allows for a controllable worm burden, consistent infection timing and more thorough clearance after treatment.

Notably, in our study, while the egg-derived sja-miR-7-5p also turned negative after PZQ treatment, its levels frequently hovered near the cut-off value, in contrast to the more pronounced and sustained decrease observed for the adult worm-derived sja-miR-61. This discrepancy may be attributed to the fact that PZQ is primarily effective against adult worms but not less mature eggs. Although eggs may persist in host tissues after treatment, deceased adult worms cease miRNA production. The remaining entrapped eggs might continue to release exosome miRNAs for a limited time. Consequently, following PZQ treatment, serum levels of adult exosome-derived sja-miR-61 decline sharply, whereas egg exosome-derived sja-miR-7-5p, though decreased, may more frequently approach the cut-off, potentially leading to ambiguous results. These findings suggest that parasite exosome-derived sja-miR-61 holds greater promise for monitoring treatment response.

Accurate diagnosis of low-intensity schistosome infections is crucial, particularly among travelers from endemic regions who may act as potential sources of transmission if undetected [67]. Thus, developing sensitive diagnostic methods for low-level infections is a research priority. Studies by Jing Xu et al. successfully detected *S. japonicum* DNA in sera of rabbits with low-intensity infections (defined as fewer than 10 eggs per gram of feces) and reported high sensitivity (95.1%) in human low-infection cohorts [68], supporting the utility of nucleic acid-based tests in such settings. RT-PCR can detect specific miRNAs at concentrations as low as 0.1–10 fmol [69]. In our study, both sja-miR-61 and sja-miR-7-5p were detectable in mice infected with only five cercariae (mean worm burden: 2.0 ± 2.1). At this minimal infection level, three out of five mice tested positive for each miRNA, corresponding to a sensitivity of 60%. This confirms the diagnostic relevance of both miRNAs even in very low-intensity infections. Although the infection level is speculative based on data from mice, it provides a useful guideline for human low-infection detection. In the future, it will be necessary to verify the low-level infection in the human population.

The limitations of our study are the relatively small number of patient samples and the long storage duration of human serum samples (up to 10 years), which may have contributed to the lower sensitivity of circulating miRNA detection in human subjects compared to the mouse model. As China has achieved interruption of schistosomiasis transmission and progresses toward elimination, acquiring fresh patient serum samples has become increasingly challenging. Furthermore, the performance of the assay for diagnosing low-intensity and early infections in human populations could not be comprehensively validated. Additionally, the precision of the sensitivity and specificity estimates was constrained by the limited number of cases. Accordingly, the 95% confidence intervals for the sensitivity and specificity of the miRNAs ranged from approximately 30% to 40%. Therefore, larger and independent cohorts are required to validate these findings and to provide more precise estimates of diagnostic accuracy to support clinical translation.

Future studies should aim to collect more fresh serum samples from infected patients, as well as from wildlife reservoir hosts and individuals with other parasitic infections, to further validate the diagnostic utility of sja-miR-61 and sja-miR-7-5p in both human and animal schistosome infections. In this study, specificity was evaluated solely using healthy controls. Since serum samples from individuals infected with other helminths (e.g., Ascaris, Strongyloides, or filarial nematodes) were not tested, the true clinical specificity of the assay remains to be determined. Future studies incorporating a broader panel of pathogen-specific controls are needed to fully establish the specificity of these schistosomal miRNA markers.

Overall, our study suggests that miRNAs derived from parasite exosomes, serve both as circulating biomarkers for parasites infection, even in low-level infection, and evaluation of drug efficacy in treatment. The results of our study will provide an effective diagnostic method for patients and animals with schistosome infection.

## Supporting information

S1 Table The primer sequences of the top 9 miRNAs and housekeeping gene U6 expressed in *S. japonicum* cercaria, adults and eggs. (DOCX)

S2 Table miRNAs obtained from *S. japonicum* worms by sequencing. (XLS)

S3 Table miRNAs obtained from *S. japonicum* eggs by sequencing. (XLS)

S1 Fig The relative expression of miRNAs in the cercariae, adult worms, and egg stages of schistosome. (DOCX)

S2 Fig The sequencing results of the miRNA amplification products from infected mice serum. (DOCX)

S3 Fig The amplification curve after adding miRNA to human serum. (DOCX)
