# Supplementary material for: Parasite exosomes-derived circulating sja-miR-61 and sja-miR-7-5p as Novel biomarkers for the detection of Schistosoma japonicum infection using TaqMan real-time PCR
Source: PLoS Negl Trop Dis. 2026 May 20;20(5):e0014368. doi: 10.1371/journal.pntd.0014368 (PMC13218617; doi:10.1371/journal.pntd.0014368)
Supplement: S1 Table — (DOCX) [file pntd.0014368.s001.docx]

**S1 Table.**

**The primers sequences of the top 9 miRNAs and housekeeping gene U6 expressed in *S. japonicum* cercaria, adults and eggs.**

| **miRNAs** | **Primers** | **Sequences** |
| --- | --- | --- |
| **sja-miR-125b** | Stem-loop | GTCGTATCCAGTGCAGGGTCCGAGGTATTCGCACTGGATACGACGAGCAA |
|  | Forward | CGCGTCCCTGAGACTGATAA |
|  | Reverse | AGTGCAGGGTCCGAGGTATT |
| **sja-miR-71a** | Stem-loop | GTCGTATCCAGTGCAGGGTCCGAGGTATTCGCACTGGATACGACTCTCAC |
|  | Forward | GCGCGTGAAAGACGATGGTA |
|  | Reverse | AGTGCAGGGTCCGAGGTATT |
| **sja-miR-125** | Stem-loop | GTCGTATCCAGTGCAGGGTCCGAGGTATTCGCACTGGATACGACAGCAAT |
|  | Forward | GCGCGTCCCTGAGACTGATA |
|  | Reverse | AGTGCAGGGTCCGAGGTATT |
| **sja-miR-61** | Stem-loop | GTCGTATCCAGTGCAGGGTCCGAGGTATTCGCACTGGATACGACAAGTGA |
|  | Forward | GCGCGTGACTAGAAAGTGCAC |
|  | Reverse | AGTGCAGGGTCCGAGGTATT |
| **sja-miR-36-3p** | Stem-loop | GTCGTATCCAGTGCAGGGTCCGAGGTATTCGCACTGGATACGACGCGAAT |
|  | Forward | CGCCACCGGGTAGACATTC |
|  | Reverse | AGTGCAGGGTCCGAGGTATT |
| **sja-miR-1** | Stem-loop | GTCGTATCCAGTGCAGGGTCCGAGGTATTCGCACTGGATACGACGACCAT |
|  | Forward | GCGTGGAATGTGGCGAAGT |
|  | Reverse | AGTGCAGGGTCCGAGGTATT |
| **sja-miR-71b-5p** | Stem-loop | GTCGTATCCAGTGCAGGGTCCGAGGTATTCGCACTGGATACGACCGTCTC |
|  | Forward | GCGCGTGAAAGACTTGAGTAGT |
|  | Reverse | AGTGCAGGGTCCGAGGTATT |
| **sja-bantam** | Stem-loop | GTCGTATCCAGTGCAGGGTCCGAGGTATTCGCACTGGATACGACACCAGC |
|  | Forward | CGCGTGAGATCGCGATTAAA |
|  | Reverse | AGTGCAGGGTCCGAGGTATT |
| **sja-miR-7-5p** | Stem-loop | GTCGTATCCAGTGCAGGGTCCGAGGTATTCGCACTGGATACGACAACAAC |
|  | Forward | CGCGTGGAAGACTGGTGATAT |
|  | Reverse | AGTGCAGGGTCCGAGGTATT |
| **U6** | Forward | GCTTCGGCAGCACATATACTAAAAT |
|  | Reverse | CGCTTCACGAATTTGCGTGTCAT |
