# Supplementary material for: Parasite exosomes-derived circulating sja-miR-61 and sja-miR-7-5p as Novel biomarkers for the detection of Schistosoma japonicum infection using TaqMan real-time PCR
Source: PLoS Negl Trop Dis. 2026 May 20;20(5):e0014368. doi: 10.1371/journal.pntd.0014368 (PMC13218617; doi:10.1371/journal.pntd.0014368)
Supplement: S1 Fig — (DOCX) [file pntd.0014368.s004.docx]

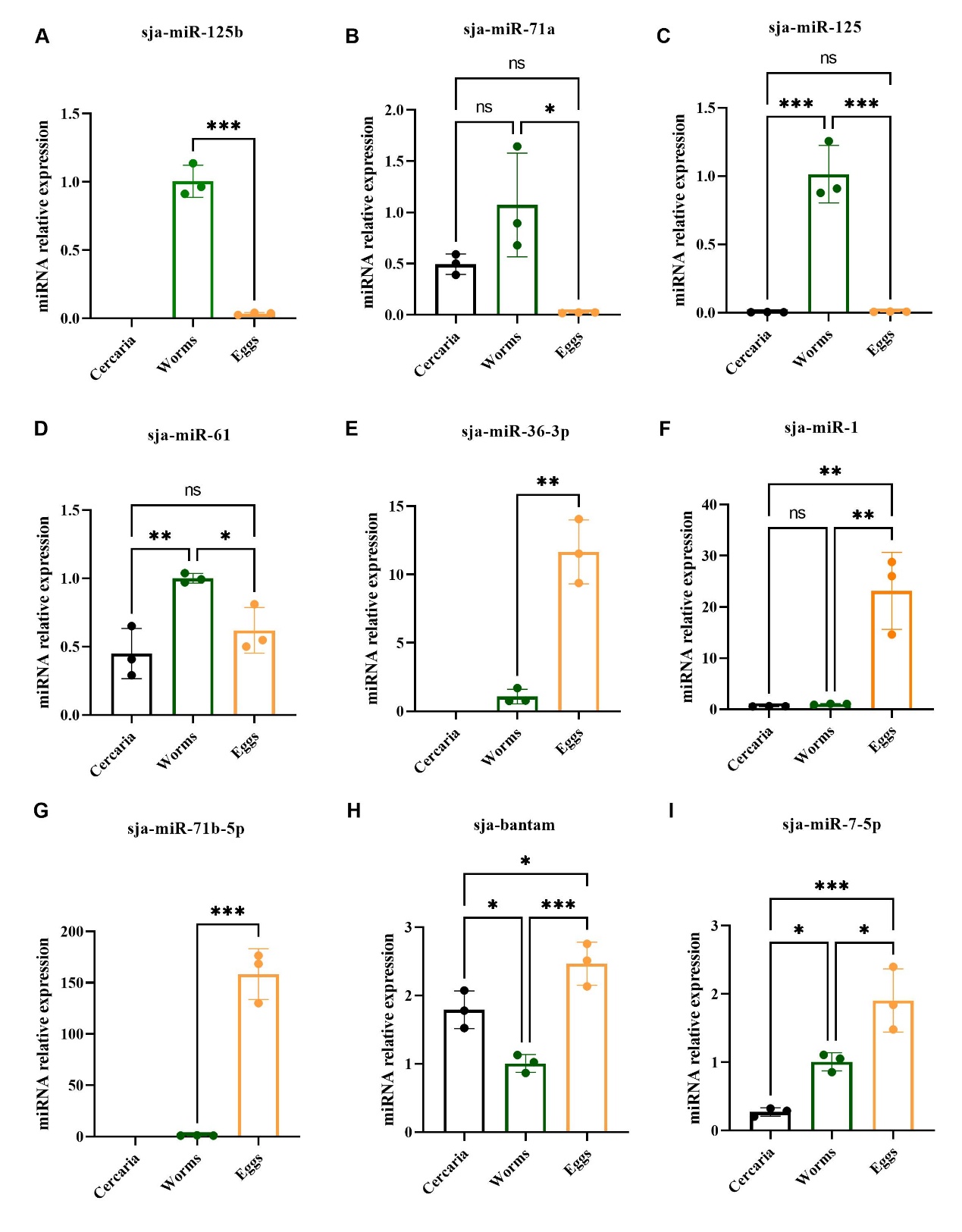


S4 Figure. The relative expression of miRNAs in the cercariae, adult worms, and egg stages of schistosome. A sja-miR-125b; B sja-miR-71a; C sja-miR-125; D sja-miR-61; E sja-miR-36-3p; F sja-miR-1; G sja-miR-71b-5p; H sja-bantam; I sja-miR-7-5p (**p*<0.05, ** *p* < 0.01, *** *p* < 0.001).
