# Supplementary figures and images for: Parasite exosomes-derived circulating sja-miR-61 and sja-miR-7-5p as Novel biomarkers for the detection of Schistosoma japonicum infection using TaqMan real-time PCR
Source: PLoS Negl Trop Dis. 2026 May 20;20(5):e0014368. doi: 10.1371/journal.pntd.0014368 (PMC13218617; doi:10.1371/journal.pntd.0014368)

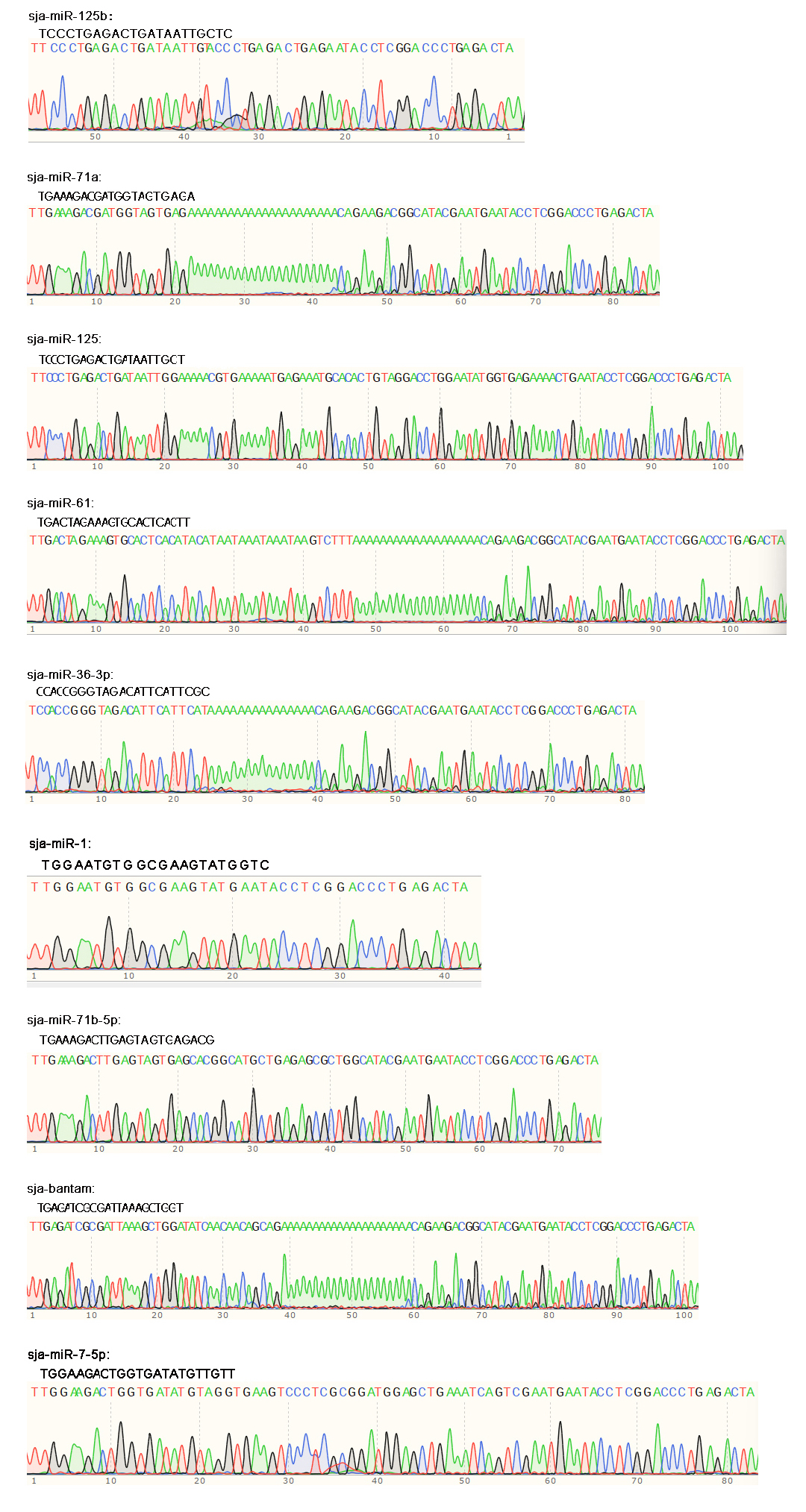


S5 Figure. The sequencing results of the miRNA amplification products from infected mice serum.

Supplement: S2 Fig — (DOCX) [file pntd.0014368.s005.docx]
