# Supplementary material for: Parasite exosomes-derived circulating sja-miR-61 and sja-miR-7-5p as Novel biomarkers for the detection of Schistosoma japonicum infection using TaqMan real-time PCR
Source: PLoS Negl Trop Dis. 2026 May 20;20(5):e0014368. doi: 10.1371/journal.pntd.0014368 (PMC13218617; doi:10.1371/journal.pntd.0014368)
Supplement: S3 Fig — (DOCX) [file pntd.0014368.s006.docx]

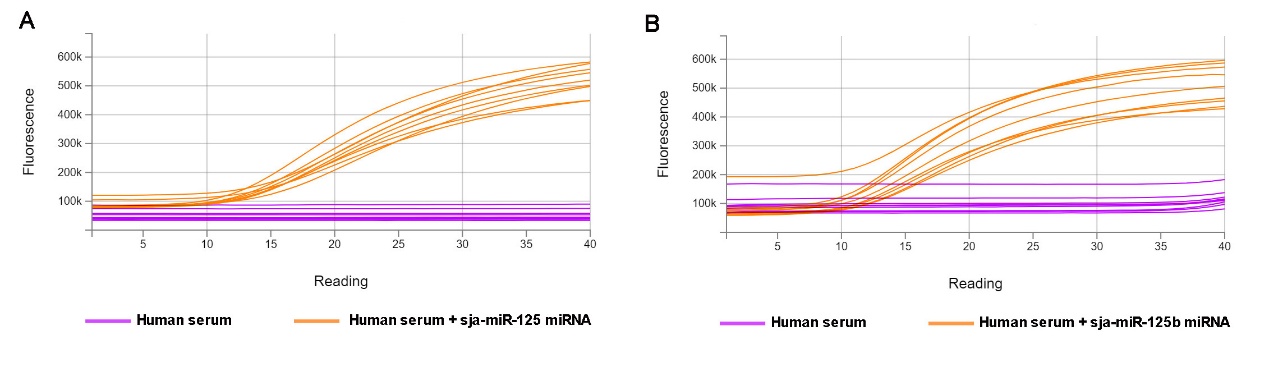


S6 Figure. The amplification curve after adding miRNA to human serum. A The amplification curve after adding sja-miR-125 to human serum; B The amplification curve after adding sja-miR-125b to human serum.
